# P300 Event-Related Potentials Mediate the Relationship Between Child Physical Abuse and Externalizing Behavior

**DOI:** 10.3389/fpsyg.2021.720094

**Published:** 2021-11-01

**Authors:** Naixue Cui, Adrian Raine, Cynthia A. Connolly, Therese S. Richmond, Alexandra L. Hanlon, Catherine C. McDonald, Jianghong Liu

**Affiliations:** ^1^School of Nursing and Rehabilitation, Shandong University, Jinan, China; ^2^Department of Family and Community Health, School of Nursing, University of Pennsylvania, Philadelphia, PA, United States; ^3^Department of Criminology, Psychiatry and Psychology, School of Arts & Sciences, University of Pennsylvania, Philadelphia, PA, United States; ^4^Department of Biobehavioral Health Sciences, School of Nursing, University of Pennsylvania, Philadelphia, PA, United States; ^5^Department of Statistics, Virginia Tech, Blacksburg, VA, United States

**Keywords:** physical abuse, P300 amplitude, event-related potential, externalizing behavior, mediation

## Abstract

The psychophysiological mechanism linking early childhood experiences to behavior problems remains unclear. This study aimed to examine the association of child physical abuse with P300 event-related potentials (ERP), and to test the mediating effect of P300 amplitude and latency in the relationship between child physical abuse and externalizing behaviors. Cross-sectional secondary data were obtained from 155 children (55.5% boys, mean age: 11.28 ± 0.57 years) who participated in the China Jintan Child Cohort Study. Children self-reported maternal and paternal physical abuse and externalizing behaviors, as well as P300 were obtained in 2013. Additionally, parents and teachers reported child externalizing behaviors in preschool in 2007. P300 were recorded during a standard novel auditory oddball task. Path analysis shows that after controlling for child sex, socioeconomic status, area of residence, IQ, and child externalizing behavior in preschool, children exposed to maternal physical abuse exhibited increased novelty P300 amplitude, which links to more externalizing behavior. Novelty P300 amplitude partially mediated the relationship between maternal physical abuse and externalizing behavior. These findings are the first to document the partial mediating effect of P300 amplitude on the abuse-externalizing relationship and are consistent with the view that physical abuse affects the attention bias to novel cues that likely places them at increased risk for the development and maintenance of externalizing behavior.

## Introduction

The relationship between child physical abuse and behavior problems has been well documented in the literature across cultures (Fry et al., 2012; Gershoff et al., 2012; Pace et al., 2019). Consistently, a recent meta-analytic study of 42 studies in Mainland China also found that physically abused children manifested more externalizing behaviors (Cui and Liu, 2020). The link remains significant even after controlling for other forms of maltreatment (Renner and Boel-Studt, 2017; Muniz et al., 2019), and the effect of physical abuse practiced by mothers demonstrates more salient effect on externalizing behaviors than that practiced by fathers (Cui et al., 2018).

Nevertheless, not all maltreated children develop behavior problems, which raises the question of the mechanism underlying the association between child physical abuse and behavior problems. Altered neurocognitive development related to child physical abuse is suggested to be a potential pathway (McCrory et al., 2012; Kavanaugh et al., 2017), which is supported by the empirical evidence of the mediating effect of neurocognition measured by neuropsychological tasks or functional magnetic resonance imaging (fMRI) in the relationship between child maltreatment and externalizing behavior (Xing et al., 2018; Hallowell et al., 2019).

At the neurophysiological level, the P300 event-related potential (ERP) is a widely-used proxy measure of the allocation of neural resources and neurocognitive processing capability (Polich, 2007). The P300 is a large positive-going peak occurring approximately 300 to 800 ms following stimulus onset. A three-stimulus oddball paradigm is widely used for elicitation of P300, in which participants are instructed to detect infrequent deviant stimuli (target; e.g., low-pitched tone) amongst a series of standard stimuli (non-target; e.g., high-pitched tone) and novel stimuli (e.g., dog barks and bird chirp) (Gao et al., 2011). Two P300 components, the “P3a” and “P3b” can be derived from such a task to assess the participants’ neural processes of directing attention to events of importance. The P3a is elicited by novel stimuli (i.e., stimuli with low probability, task-irrelevant, but contextual salience) and is commonly regarded as reflecting a bottom-up process of attention orienting to prepare the organism for deviant events in the environment (Debener et al., 2002; Polich, 2007). The P3b peaks 60–80 ms later than the P3a, and is considered a measure of effortful top-down attentional shift to infrequent but task-relevant stimulus and working memory updating when participants are actively engaged in the task of detecting the targets (Polich, 2007). P300 has been extensively studied in relation to both child maltreatment and externalizing behavior, respectively.

A number of studies have examined the effects of maltreatment on neural processes utilizing ERP. For example, Pollak et al. (2001) have conducted a series of studies on school-age maltreated children and found that maltreated children show hyper-responsive to angry facial expressions and greater P3b amplitude in response to angry facial affect (Pollak and Tolley-schell, 2003; Shackman et al., 2007; Shackman and Pollak, 2014). These findings indicate that enhancement in P3b amplitude may be an adaptive mechanism to help maltreated children to detect and respond to anger stimuli more efficiently (Shackman and Pollak, 2014).

Literature also documents relatively consistent evidence that an attenuated P300 amplitude is an endophenotype of externalizing disorders featured by excessive impulsivity (Patrick et al., 2006; Brennan and Baskin-Sommers, 2018), such as alcohol use disorder and substance disorders (Iacono and Mcgue, 2002; Euser et al., 2012; Hamidovic and Wang, 2019), antisocial behavior and impulsive-antisocial psychopathy (Pasion et al., 2018), attention deficit hyperactivity disorder (ADHD; Bitter, 2011; Kallen et al., 2020), and conduct disorder (Iacono and Mcgue, 2002). Reduced novelty P300 amplitude in oddball tasks using non-affective auditory or visual stimuli was found among criminal psychopaths (Venables and Patrick, 2014), offenders (Brazil et al., 2012), and men convicted of spousal/partner abuse (Stanford and Kockler, 2007) in comparison to healthy controls. P300 amplitude reduction also shows association with deficient inhibit control, a cognitive process that links to externalizing behavior.

Despite the cumulative evidence of the pairwise relationships among child maltreatment, P300, and externalizing behavior, the more complex relationships among the three constructs have not been fully examined. To our best knowledge, only two studies (Shackman et al., 2007; Shackman and Pollak, 2014) have hypothesized the pathway leading maltreatment to behavior problems through impaired neural activity indicated by P300 abnormalities and tested the hypothesis empirically. Shackman et al. (2007) in a study of 30 male and female children reported that physically abused children exhibited increased P300 amplitude to threatening stimuli (i.e., their mother’s angry faces and angry voices), and that enhanced P300 amplitude mediated the relationship between physical abuse and child self-reported anxiety. Findings from a subsequent study of 50 boys by Shackman and Pollak (2014) found that physical abuse was associated with increased P300 amplitude to negative visual stimuli (i.e., angry adult faces), but that increased P300 amplitude did not significantly correlate with child self-reported aggression.

The inconsistent findings in these studies may be attributed to the differences in participant characteristics (e.g., both boys and girls vs. boys only and age differences), task modality (both visual and vocal stimuli from children’s mothers vs. visual stimuli from unfamiliar adults), and behavioral outcomes (anxiety reported by parents using a questionnaire vs. aggression measured objectively using an aggression task). Moreover, the non-significant finding in Shackman and Pollak (2014) may be due to lack of statistical power since the sample size was small (*N* = 50). In addition, neither study controlled for possible confounders, such as prior behavior problems in the analysis. Therefore, larger-scale studies using a standard task protocol to elicit P300 are necessary to further investigate the relationships among physical abuse, P300, and child externalizing behavior.

The objectives of this study were twofold: (1) to test the relationship between child physical abuse and P300 amplitude elicited by a standard novel auditory oddball task stimuli, and (2) to examine the mediating effect of P300 amplitude to novel and target stimuli in the relationship between child physical abuse and externalizing behavior. Because prior studies have not controlled for the possibility that earlier externalizing behavior could result in later enhanced P300 (as opposed to enhanced P300 predisposing to externalizing behavior), we controlled for externalizing behavior collected 6 years prior to P300 assessment.

## Materials and Methods

### Design and Participants

This is a cross-sectional study using secondary data (de-identified) collected from a sub-cohort of children during Wave II (T2) of the China Jintan Child Cohort Study (the Jintan Study). The Jintan Study is an ongoing prospective longitudinal study to investigate the impact of environmental exposures, such as lead, on children’s neurobehavioral outcomes (Liu et al., 2010, 2015), and child physical abuse was measured as an important social confounder. It initially recruited three sub-cohorts of children when they were 3 to 5 years old in 2004–2005. All of these children were invited to participate in two waves of data collection when they were about 6 years old in preschool in 2005 (sub-cohort 1), 2006 (sub-cohort 2), and 2007 (sub-cohort 3), and when they were about 12 years old in grade 6 elementary school in 2011 (sub-cohort 1), 2012 (sub-cohort 2), and 2013 (sub-cohort 3), respectively. The cohort children were regarded representative of children of the same age in Jintan City, a small-scale city on the east coast in Mainland China. Details of the cohort design and sampling information are described elsewhere (Liu et al., 2010, 2011, 2015).

In 2013, in additional to the T2 questionnaire survey, all the children of sub-cohort 3 (*n* = 414) were also invited to participate in psychophysiological recordings. Out of the 414 children, 155 with complete data on child physical abuse, P300, and externalizing behavior at T2 were included in this study. Comparisons of these children and the remaining sub-cohort children who were not included in this study demonstrated no significant differences in age, sex, socioeconomic status, area of residence, externalizing behaviors at age 6 and 12, and maternal and paternal physical abuse experiences. Table 1 displays the sample characteristics and the comparison results.

**TABLE 1:** Sociodemographic characteristics of the present sample and comparisons with the children not included in the present study.

	**Children included (*n* = 155) M ± SD/*n*(%)**	**Children excluded (*n* = 259) M ± SD/*n*(%)**	***t/*χ^2^**	***p-*Value**
Age (*n*_i_ = 155, *n*_e_ = 257)	11.28 ± 0.57	11.32 ± 0.54	0.71	0.48
Gender			0.01	0.98
Girls	69 (44.5)	115 (44.4)		
Boys	86 (55.5)	144 (55.6)		
Family location			0.20	0.91
Urban	66 (42.6)	113 (43.6)		
Suburban	65 (41.9)	110 (42.5)		
Rural	24 (15.4)	36 (13.9)		
SES (*n*_i_ = 133, *n*_e_ = 140)	0.19 ± 0.99	0.22 ± 0.98	0.24	0.81
IQ (*n*_i_ = 113, *n*_e_ = 91)	105.65 ± 11.39	103.40 ± 13.09	1.31	0.19
Externalizing behavior at age 12 (*n*_i_ = 155, *n*_e_ = 184)	52.52 ± 11.43	51.18 ± 9.67	1.11	0.27
Externalizing behavior at age 6 (*n*_i_ = 132, *n*_e_ = 218)	13.23 ± 6.74	13.47 ± 6.96	0.31	0.76
Maternal physical abuse			2.52	0.11
Yes	51 (32.9)	37 (24.7)		
No	104 (67.10)	113 (75.3)		
Paternal physical abuse			1.24	0.27
Yes	54 (37.8)	47 (29.0)		
No	101 (65.2)	115 (71.0)		

*SES, socioeconomic status; IQ, intellectual quotient. Family location was self-identified. *n*_i_, number of children with available information included in this study; *n*_e_, number of children with available information but *not* included in this study.*

We obtained verbal assent from children and informed consent from their parents. This study was approved by the Institutional Review Board (IRB) of the University of Pennsylvania and the Ethics Committee of the Jintan Hospital. Clear instruction of voluntary participation, rights of withdrawing or skipping questions whenever they did not feel like answering was given to the children. Considering the potential distress caused by completing the survey, especially questions regarding harsh parenting practice, information of school psychological services and local professional mental health institutions were provided for all participated children in case they needed. When the study was conducted, there was no mechanism/laws/regulations of reporting and dealing with cases of physical abuse that did not meet the criteria of crime in Mainland China. Therefore, action of reporting was not taken.

### Measures

#### Child Physical Abuse at Age 12 Years Old

Children reported their physical abuse experiences in the previous year using the severe physical assault subscale of the Chinese Version of the Parent–Child Conflict Tactics Scale (CTSPC; Straus and Hamby, 1998). They were asked to provide information on whether they were: (1) hit on body parts besides the bottom with objects, (2) thrown or knocked down, (3) hit with a fist or kicked hard, (4) beaten up, (5) grabbed around the neck and choked, (6) burned or scalded on purpose, or (7) threatened with a knife or other weapons by their mothers and fathers separately in the preceding year (0 = “No”, or 1 = “Yes”). Children who answered “Yes” to at least one of these items were regarded as having experienced physical abuse. The CTSPC has shown good construct validity (Straus and Hamby, 1998) and reliability in Chinese studies (Chan, 2012; Cui et al., 2016, 2018). In the present study, Cronbach’s alpha coefficients for maternal (0.84) and paternal (0.87) physical abuse were acceptable. Our previous study using the same data source demonstrated that children with only maternal physical abuse scored higher on behavior problems but those with only paternal physical abuse did not, and the effect of both maternal and paternal physical abuse on behavior problems was driven by maternal physical abuse. Therefore, we tested the effect of maternal and paternal physical abuse separately, even though they could co-occur.

#### Child Externalizing Behaviors at Age 12 and 6 Years Old

Externalizing behaviors at ages 12 and 6 were reported by children themselves using the Chinese version of the Youth Self-Report (YSR) and by mothers using the Chinese version of Child Behavior Checklist for age 1.5–5 (CBCL/1.5–5), respectively. Each item was evaluated on a 3-point scale (2: “often true”, 1: “sometimes”, and 0: “not true”). The sum score of all items in the externalizing behavior subscale were normalized (mean = 50.00 and standard deviation = 10.00) to obtain *T* scores for data analysis according to the measurement manuals (Achenbach and Rescorla, 2000). Higher *T* scores indicate more externalizing behavior. The YSR and CBCL showed good reliability and validity in Chinese children and adolescents (Su et al., 1998; Wang et al., 2013). Complete data on externalizing behavior at age 12 and 6 were obtained from 279 children and 350 mothers, respectively.

#### Psychophysiological Assessment at Age 12 Years Old

##### Standard novel auditory oddball task

Given the overarching goal of the parent study of investigating the impact and mechanism of environmental exposure on child neurobehavioral outcomes, the standard novel auditory oddball paradigm was chosen because it was widely used in studies of brain function and behavioral problems with externalizing traits [e.g., substance abuse (Euser et al., 2012; Hamidovic and Wang, 2019), and psychopathology (Gao et al., 2018)]. The oddball task used in this study contains 280 high-pitched tones (non-target, presented at 1000 Hz) and 35 low-pitched tones (target, presented at 500 Hz), as well as 35 novel tones (e.g., dog-bark, bell, bird, and honk) at 75 dB. Each tone lasted for 150 ms, with an inter-stimulus interval of 1.1 s, an inter-trial interval of 1.25 s, and rise and fall times of 5 ms. The target, non-target, and novel tones were presented in random order. The duration of the task was 7.5 min.

Children were tested in a temperature-controlled, light- and sound-attenuated laboratory, with a computer screen placed at a distance of 1 m. For the duration of the task, the children were instructed to keep their eyes fixated on an “X” on the computer screen. To ensure they could distinguish between the non-target and target tones before the actual test, they were given six practice trials. In the actual test, they were instructed to press a response button as quickly as possible with their dominant hand in response to the target tones, but not to other tones. The number and reaction time of correct responses to target, and the number of incorrect responses to non-target (commission error) and novel stimuli (false alarms) were recorded as behavioral information-processing indicators.

##### Event-related potentials recording and data acquisition

During the oddball task, electroencephalography (EEG) was recorded from an Electro-Cap (Eaton, OH, United States) with tin (Sn) electrodes placed at 12 sites on the scalp (FP1, FP2, F3, F4, F7, F8, P3, P4, T3, T4, O1, and O2) according to the International 10–20 system. A single-channel EEG100C biopotential module (BIOPAC Systems, Inc., Goleta, CA, United States) was used to amplify the EEG signal from each electrode. The same parameters used in Rudo-Hutt’s (2014) study were applied. Specifically, the EEG signal was grounded *via* 8 mm diameter silver/silver chloride (Ag/AgCl) electrodes attached to the distal phalanges of the first and second fingers of the non-dominant hand. In addition, an electrooculograph (EOG) channel monitored vertical eye movement *via* 4 mm diameter Ag/AgCl electrodes placed above and below the supra- and infra-orbital ridges of the left eye. A Q-tip stick was used to abrade the scalp electrode sites, whereas skin on the earlobes and around the left eye was prepared using NuPrep abrasive skin prepping paste. Biopac isotonic recording gel was used as the electrolyte medium for EOG, and Electro-gel was used for the earlobes and scalp. Impedance for EEG was kept below 10 kΩ and was under 5 kΩ for most participants, while impedance for EOG and ear electrodes was kept below 20 kΩ. Data from EEG channels were recorded using a bandpass of 0.01–35 Hz and a 50 Hz notch filter, with a 1000 Hz sampling rate and gain set to 5000. Data from the EOG channel were recording using a bandpass of 0.05–35 Hz and a 50 Hz notch filter, with a 1000 Hz sampling rate and gain set to 1000.

After ERP recording, data from each EEG channel were visually inspected in Acq*Knowledge* (BIOPAC Systems Inc., Goleta, CA, United States), and clearly artifactual data on EEG (e.g., due to equipment failure or eye movement) were discarded. To help better ensure fidelity of data, the EEG was further processed for remaining artifacts by rejecting EEG epochs that exceeded ±80 μV using custom scripts in MATLAB (MathWorks Inc., Natick, MA, United States). Next, the cleaned EEG data was divided into epochs based on stimulus presentation (from 200 ms before to 800 ms after each stimulus) and averaged over all trials and all electrodes for each stimulus type in MATLAB to generate the average P300 (i.e., the greatest positive deflection post-stimulus) amplitude across electrode sites to target, non-target, and novel stimuli, respectively. P300 was defined as the largest positive-going wave in the range of 100–600 ms after the stimulus onset. A total of 166 children completed the ERP recording.

#### Other Covariates

In addition to mother-reported externalizing behavior at age 6, other covariates include child sex, socioeconomic status (SES), area of residence (i.e., urban, suburban and rural areas reported by mothers) at the time of cohort recruitment, and intellectual functioning (IQ) at age 12 because these variables were both associated with physical abuse and externalizing behaviors based on literature (Fergusson and Horwood, 2002; Liao et al., 2011; Cui and Liu, 2016) and data availability of the parent study. SES was calculated as the standardized *z* score of the sum of standardized *z* scores of mothers’ and father’s education years and monthly wage as described in Straus (2004). IQ was measured using the validated Chinese version of the Wechsler intelligence scale for children-revised (WISC-R; Dan and Yu, 1990; Liu and Lynn, 2015).

### Statistical Analysis

Complete data were obtained on key variables (physical abuse, P300, and externalizing behavior at age 12 years old) from 159 children. Four children were further excluded because one had an IQ lower than 70 and three had 0 correct responses to targets in the oddball task. Therefore, data from 155 children were used in further analysis.

Sample characteristics of the 155 children were compared with their counterparts who were not included. Bivariate analyses, including independent *t*-tests, Wilcoxon sum rank test, *Pearson* correlation were used to examine the bivariate association among physical abuse, externalizing behavior, behavioral performances on the oddball task, and target P300 (P3b) and novelty P300 (P3a) amplitude. The P300 variables that showed bivariate relationships with physical abuse or externalizing behavior with *p-*values less than 0.25 were submitted to the path analysis as potential mediators (Bursac et al., 2008).

**FIGURE 1:**
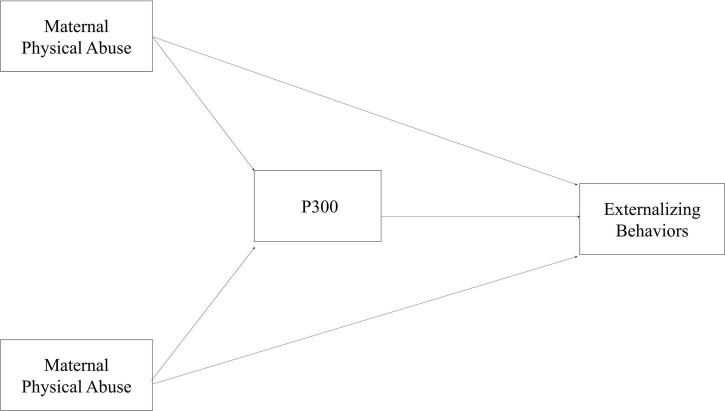
The full mediation model. The model illustrates the relationship between maternal and paternal physical abuse and self-reported externalizing behaviors and the mediating role of P300. The variable P300 refers to any of the following variables: novelty P300 amplitude and target P300 amplitude. All the variables in the figure were measured at age 12. The model was adjusted child sex, socioeconomic status, area of residence, IQ at age 12, anxiety at age 12 and externalizing behaviors in preschool at age 6.

Path analysis using structural equation modeling (SEM) was implemented to analyze the mediating effect of P300 amplitude based on the model shown in Figure 1. Maternal and paternal physical abuse served as the initial exogenous variables with direct paths to externalizing behavior and indirect paths to externalizing behavior through the P300 variable(s) identified from the above process, controlling for the covariates. The full information maximum likelihood method was used to address missing data on mother-reported externalizing behavior at age 6. Because a mediation model is saturated, the commonly used goodness of fit indices, such as root square error of approximation (RMSEA), comparative fit index (CFI) and Tucker-Lewis Index (TFI) cannot be applied. Instead, as recommended by Kenny (2016), Akaike Information Criterion (AIC) and Bayesian Information Criterion (BIC) were used to evaluate the mediation model fit by comparing the model without the direct path, the model without the path from predictor to mediator, and the model without the path from mediator to the outcome, respectively. The model with the smallest AIC or BIC was selected. Bootstrapping with 500 replications was used to estimate the bias-corrected 95% confidence interval for the indirect, direct and total effects. Significance level was set at α = 0.05. Analyses were performed using STATA 13.0 for Windows (College Station, TX, United States).

## Results

### Sample Characteristics

There were slightly more boys (86, 55.5%) than girls (69, 44.5%) among the included sample. The average age was 11.28 ± 0.57 years old. A total of 51 (32.9%) children reported maternal physical abuse, and 54 (37.8%) reported paternal physical abuse, out of whom 41 reported physical abuse by both mothers and fathers. See Table 1.

### Bivariate Associations of Physical Abuse With Behavioral Performance on the Oddball Task, P300 and Externalizing Behavior

Children who experienced physical abuse by either mother or father displayed significantly more externalizing behavior at age 12 when compared with their non-abused counterparts (Table 2). Children with maternal physical abuse showed increased novelty P300 amplitude (11.40 ± 4.30 μV) compared with non-maternal-abused counterparts (9.63 ± 4.47 μV), but the associations of paternal physical abuse with P300 parameters did not yield any statistical significance. Neither maternal nor paternal physical abuse showed significant associations with the behavioral performances on the oddball task.

**TABLE 2:** Comparisons of behavior problems, performance on oddball task and P300 between physically abused children and their non-abused counterparts.

	**Maternal physical abuse**				**Paternal physical abuse**			
	**No M ± SD(Median)**	**Yes M ± SD(Median)**	***t/z***	***p***	**No M ± SD(Median)**	**Yes M ± SD(Median)**	***t/z***	***p***
Externalizing behavior at age 12	50.60 ± 9.24	56.43 ± 14.24	3.07	<0.001	51.09 ± 11.85	55.18 ± 10.15	2.15	0.03
Externalizing behavior at age 6	12.84 ± 6.91	14.12 ± 6.34	1.02	0.31	12.68 ± 6.54	14.43 ± 7.08	1.40	0.17
Mean reaction time	504.07 ± 97.5	482.64 ± 78.09	1.36	0.18	501.05 ± 92.97	489.56 ± 90.25	0.73	0.46
Correct response to target	31.38 ± 4.22 (33)	30.58 ± 5.35 (32)	0.73*^*w*^*	0.46	31.32 ± 4.1 (32)	30.74 ± 5.48 (33)	0.11*^*w*^*	0.91
Errors to novels	5.63 ± 7.44 (3)	5.54 ± 6.95 (3)	0.21*^*w*^*	0.83	6.16 ± 8.09 (3)	4.55 ± 5.25 (3)	0.23*^*w*^*	0.82
Errors to non-targets	1.59 ± 2.05 (1)	1.94 ± 1.97 (1)	1.45*^*w*^*	0.15	1.47 ± 1.81 (1)	2.15 ± 2.34 (1)	1.88*^*w*^*	0.06
Novelty P300 amplitude (μV)	9.63 ± 4.47	11.40 ± 4.30	2.35	0.02	10.04 ± 4.66	10.54 ± 4.14	0.67	0.51
Target P300 amplitude (μV)	10.98 ± 7.13	11.08 ± 5.06	0.09	0.93	11.31 ± 7.15	10.46 ± 5.09	0.77	0.44

*The parenthetical numbers in the 2nd, 3rd, 5th and 6th columns refer to the medians of the variables among corresponding subgroup of children. Superscript *w*, Wilcoxon rank-sum test.*

### Relationship Between Externalizing Behavior at 12 and 6 Years old and P300

Externalizing behavior at 12 and 6 years old were positively correlated with a small correlation coefficient (*r* = 0.204, *p* = 0.020). More externalizing behavior at 12 years old was positively correlated with higher novelty P300 amplitude (*r* = 0.255, *p* < 0.01), but was not significantly correlated with target P300 amplitude (*r* = 0.028, *p* = 0.728). See Table 3. Therefore, novelty P300 amplitude was submitted to path analysis as a mediator and target P300 amplitude was not. Paternal physical abuse was dropped from the path model because it was significantly related to neither the mediator (i.e., novelty P300 amplitude) nor the externalizing behavior.

**TABLE 3:** Pearson correlations between ERP and behavior problem.

	**1**	**2**	**3**	**4**
1. Externalizing behavior at age 12	1			
2. Externalizing behavior at age 6	0.204*	1		
3. Novelty P300 amplitude (μV)	0.255**	0.171	1	
4. Target P300 amplitude (μV)	0.028	0.135	0.250**	1

*, *p* < 0.05; **, *p* < 0.01.

### Path Analysis Results

**FIGURE 2:**
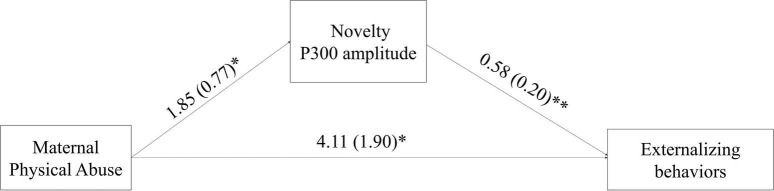
The final mediation model with path coefficients. The model illustrates that novelty P300 amplitude mediated the relationship between maternal physical abuse and self-report externalizing behaviors. ^∗^, *p* < 0.05; ^∗∗^ *p* < 0.01.

The path coefficients of the final mediation model are displayed in Figure 2. The indirect, direct and total effects with bias-corrected 95% confidence interval using the bootstrapping method were 1.08 (0.26–2.58), 4.11 (0.17–10.08), and 5.19 (1.22–11.00), respectively. The AIC and BIC values were 5004.666 and 5169.011, respectively. Next, the final model was compared with itself without the direct path (AIC = 5007.294, BIC = 5169.595), as well as without the path from maternal abuse to novelty P300 amplitude (AIC = 5008.402, BIC = 5169.703), and without the path from novelty P300 amplitude to self-report externalizing behavior (AIC = 5011.274, BIC = 5172.576), respectively. The results showed that the final model itself has the smallest AIC and BIC. Taken together, novelty P300 amplitude partially mediated the relationship between maternal physical abuse and self-report externalizing behavior. The indirect effect accounts for 20.8% of the total effect between maternal physical abuse and self-report externalizing behavior.

## Discussion

This study tested the mediation hypothesis that the P300 ERP partly underlies the relationship between physical abuse and child externalizing behavior. Children who reported physical abuse by mothers also reported higher externalizing behavior problems and showed increased amplitude of the novelty P300. Externalizing behavior was positively related to novelty P300 amplitude. Further path analysis revealed that enhanced P300 amplitude to novel stimuli partially mediated the relationship between maternal physical abuse and self-reported externalizing behavior after adjusting for child sex, socioeconomic status, area of residence, IQ, and earlier externalizing behavior. To our knowledge, findings appear to be the first to document the mediating role of P300 amplitudes on the abuse-externalizing relationship.

### The Relationships Among Physical Abuse, Novelty P300 Amplitude, and Externalizing Behaviors

This study found increased novelty P300 amplitudes in a cognitive task using non-affective auditory stimuli among children who had experienced maternal physical abuse. This result is broadly consistent with prior findings showing that abused children are hypervigilant to negative visual and vocal stimuli (Pollak et al., 2001; Pollak and Tolley-schell, 2003; Shackman et al., 2007; McCrory et al., 2012; Gabriela et al., 2014; Shackman and Pollak, 2014; Okazaki et al., 2020). We extend prior literature by illustrating that physically abused children also showed hypervigilance to novel non-affective stimuli. Taken together, physically abused children may demonstrate generalized hypervigilance and tend to orient more attentional resources involuntarily toward distractors or negative emotional stimuli in the environment. This feature may reflect an adaptive mechanism for abused children to be more capable of detecting, and hence reacting more efficiently to potential social threats in the environment (Pollak, 2015).

Further, the enhanced novelty P300 amplitude partially mediated the relationship between maternal physical abuse and externalizing behaviors, which is generally consistent with the prior findings that attention problems mediated the relationship between physical abuse and aggression in children and adolescents (Kenny, 2016), and that attention bias toward mothers’ angry faces or voices indicated by enhanced P300 amplitude mediated the association of physical abuse and anxiety (Debener et al., 2002). These data are consistent with the view that physical abuse affects the attention bias to novel cues that likely place them at increased risk for the development and maintenance of externalizing behavior (Liao et al., 2011).

However, the finding that increased novelty P300 was associated with more externalizing behaviors is contradictory to the existing findings of reduced novelty P300 responses of studies among criminals, offenders, and university students (Stanford and Kockler, 2007; Brazil et al., 2012; Venables and Patrick, 2014; Bernat et al., 2020). Nonetheless, some other studies among psychopaths reported no associations (Munro et al., 2007; Gao et al., 2011) or enhanced P300 to non-affective auditory or digital stimuli (Raine, 1987; Raine and Venables, 1988; Roberti, 2004; Gao et al., 2018). Although differences in task modality (affective vs. non-affective) can be a possible reson for the mixed findings, it is also possible that different traits of psychopaths are related to different patterns of P300 change. This is supported by the notion proposed by Pasion et al. that P300 decrement may be a neurobiological marker of externalizing dispositions of many personality and mental disorders, whereas enhanced P300 amplitude during non-affective cognitive tasks is associated with interpersonal-affective psychopathic traits (Pasion et al., 2018). Hence, our finding of positive association of novelty P300 and externalizing behavior may suggest the interpersonal-affective impairment be the potential mechanism of the link between physical abuse and externalizing behavior. It may be also possible that this result was confounded by internalizing behaviors as externalizing and internalizing behaviors tend to be comorbid (Bernat et al., 2020).

It is worth noting that the participated children were generally healthy school children without obvious or diagnosed externalizing disorders, and, therefore, their neural activity may be different from those with diagnosed or severe antisocial behavior or psychopathy in the past studies. More longitudinal studies can be conducted to further investigate whether the neural activity toward novel stimuli changes from externalizing behavior to the course of externalizing disorders.

### Target P300 and Externalizing Behaviors

Unlike the previous studies that reported reduced target P300 amplitude among individuals with generic antisocial behavior (Gao and Raine, 2009), substance abuse disorders (Iacono and Mcgue, 2002; Euser et al., 2012; Hamidovic and Wang, 2019), ADHD (Bitter, 2011), and conduct disorder (Iacono and Mcgue, 2002), we did not find a significant relationship between target P300 and externalizing behavior. It may be because the oddball task used in this study is not complex enough to reveal potential cognitive deficiency in such a sample of healthy children suggested by the finding that no significant difference in the behavioral responses in the oddball task was found between abused and non-abused children. Nonetheless, a recent meta-analysis did not find a significant mean effect of target-P300 (Go-P300) amplitude between individuals with and without ADHD (Kaiser et al., 2020). Bernat et al. (2020) found that lower novelty P300 amplitude elicited by a rotated-head visual oddball task was associated with more externalizing behavior, but target P300 amplitude was not among a sample of healthy university students. Therefore, the exact relationship between target P300 and child externalizing problems needs further investigation.

### Maternal vs. Paternal Physical Abuse

The present study did not find a significant association of paternal physical abuse with P300 and externalizing behavior in the path analysis, which is consistent with the previous findings. For example, our study among Chinese children using the same data source showed that in comparison to paternal physical abuse, maternal physical abuse showed a more salient relationship with child externalizing and internalizing behaviors (Cui et al., 2018). Gao et al. (2010) reported that paternal care was not significantly associated with psychopathy after controlling for maternal care. Likewise, a meta-analysis by Kawabata et al. (2011) conducted a review of 48 studies and found that maternal parenting stress was associated with child relational regression, whereas paternal parenting stress was not.

Previous research suggested paternal and maternal abuse may link to externalizing behavior through different neurocognitive pathways that may be not captured by the P300 in the study. For example, Xing et al. (2018) found that inhibitory control mediated the relationship between maternal corporal punishment (CP) and child externalizing behavior, and working memory mediated the relationship between paternal CP and child externalizing behavior. More studies are needed to clarify the different effects of maternal and paternal parenting behaviors on child neurophysiological and behavioral development.

### Strengths and Limitations

The study’s strength lies in testing the mediating role of P300 on the relationship between physical maltreatment and externalizing behavior adjusting for earlier measured externalizing behavior in a relatively large sample (*N* = 155). The sample size is 3–5 times larger than the two prior studies [*N* = 30 and 50 (Shackman et al., 2007; Shackman and Pollak, 2014)]. Nonetheless, causal inferences cannot be made as data was cross-sectional. Furthermore, a significant proportion of children of the original sub-cohort did not participate in the psychophysiological data collection. Although comparisons of the sociodemographic characteristics between the retained children and excluded children due to missing data or drop-out from the parent study did not yield significant differences, potential differences in unobserved characteristics cannot be ruled out. In addition, other forms of child maltreatment were not assessed, and, therefore, the effect of physical abuse on P300 and externalizing behavior may be contaminated, especially considering that multiple forms of child maltreatment tend to co-occur (Brown et al., 2019). Also, this study examined the between group (exposure or non-exposure of physical abuse) differences, but did not examine the possible within group differences (e.g., frequency, severity, and chronicity) among children exposed to physical abuse in the past year. The physical abuse and externalizing behavior information were self-reported, and may be subject to report bias and common method bias. Last but not least, although the novel auditory oddball task is widely used in the area of behavioral problems with externalizing traits, it was relatively rarely used among maltreated children. It has been suggested that P300 elicited by different cognitive modalities can be different and such differences can be informative for distinguishing cognitive patterns across different populations (Barry et al., 2009; Nan et al., 2018). Future studies can utilize the bimodal/inter-modal design of oddball task to further investigate the patterns of brain activity in the context of child maltreatment and externalizing behaviors.

## Conclusion

In conclusion, physically abused children showed increased P300 amplitude to novel stimuli, and this enhancement partially mediated the relationship between physical abuse and externalizing behavior. The findings contribute to the very sparse literature on how psychophysiological pathways underpin the relationship between physical abuse and externalizing behavior. They further suggest that attention bias to novel/negative stimuli in the environment could be targeted to potentially treat childhood externalizing behavior associated with physical abuse. Such intervention studies could further test the casual nature of associations documented in this study. The findings also shed light on future research to investigate the mechanism using more rigorous methodology, such as measuring physical abuse and externalizing behaviors using objective or multiple-informant approach, taking other forms of child maltreatment into consideration, using other paradigms for ERP recording.

## Data Availability Statement

The raw data supporting the conclusions of this article will be made available by the authors, without undue reservation.

## Ethics Statement

The studies involving human participants were reviewed and approved by the Institute Review Board of University of Pennsylvania and the Ethics Committee of Jintan Hospital. Written informed consent to participate in this study was provided by the participants’ legal guardian/next of kin.

## Author Contributions

NC: conceived of the presented idea, performed the analysis, and wrote the manuscript. JL: conceptualized the cohort study, collected the data, conceived of the presented idea, and revised the manuscript. AR, CC, TR, AH, and CM: intellectual contribution to theorizing and revising the manuscript. All authors contributed to the article and approved the submitted version.

## Conflict of Interest

The authors declare that the research was conducted in the absence of any commercial or financial relationships that could be construed as a potential conflict of interest.

## Publisher’s Note

All claims expressed in this article are solely those of the authors and do not necessarily represent those of their affiliated organizations, or those of the publisher, the editors and the reviewers. Any product that may be evaluated in this article, or claim that may be made by its manufacturer, is not guaranteed or endorsed by the publisher.
